# CaMKII Requirement for *in Vivo* Insular Cortex LTP Maintenance and CTA Memory Persistence

**DOI:** 10.3389/fphar.2017.00822

**Published:** 2017-11-14

**Authors:** Yectivani Juárez-Muñoz, Laura E. Ramos-Languren, Martha L. Escobar

**Affiliations:** División de Investigación y Estudios de Posgrado, Facultad de Psicología, Universidad Nacional Autónoma de Mexico, Mexico City, Mexico

**Keywords:** CaMKII, CTA, neocortical-LTP, memory persistence, insular cortex

## Abstract

Calcium-calmodulin/dependent protein kinase II (CaMKII) plays an essential role in LTP induction, but since it has the capacity to remain persistently activated even after the decay of external stimuli it has been proposed that it can also be necessary for LTP maintenance and therefore for memory persistence. It has been shown that basolateral amygdaloid nucleus (Bla) stimulation induces long-term potentiation (LTP) in the insular cortex (IC), a neocortical region implicated in the acquisition and retention of conditioned taste aversion (CTA). Our previous studies have demonstrated that induction of LTP in the Bla-IC pathway before CTA training increased the retention of this task. Although it is known that IC-LTP induction and CTA consolidation share similar molecular mechanisms, little is known about the molecular actors that underlie their maintenance. The purpose of the present study was to evaluate the role of CaMKII in the maintenance of *in vivo* Bla-IC LTP as well as in the persistence of CTA long-term memory (LTM). Our results show that acute microinfusion of myr-CaMKIINtide, a selective inhibitor of CaMKII, in the IC of adult rats during the late-phase of *in vivo* Bla-IC LTP blocked its maintenance. Moreover, the intracortical inhibition of CaMKII 24 h after CTA acquisition impairs CTA-LTM persistence. Together these results indicate that CaMKII is a central key component for the maintenance of neocortical synaptic plasticity as well as for persistence of CTA-LTM.

## Introduction

Learning and memory rely on long-lasting changes in synaptic efficiency within neural networks. Long-term potentiation (LTP) is a long-lasting and activity-dependent enhancement of synaptic strength that is widely expressed across the brain (Malenka and Bear, 2004; Rodríguez-Durán et al., 2011). Studies in the neocortex and hippocampus have demonstrated that training in several learning tasks drive modifications of synaptic strength (Rioult-Pedotti et al., 2000; Whitlock et al., 2006; Cooke and Bear, 2010; Liu et al., 2017; Rodríguez-Durán et al., 2017). The insular cortex (IC) is a region of the temporal neocortex implicated in the acquisition and storage of conditioned taste aversion (CTA). CTA is a well-established learning and memory paradigm in which an animal acquires aversion to a novel taste when it is associated with nausea (Bernstein and Koh, 2007; Bermúdez-Rattoni, 2014; Rivera-Olvera et al., 2016). Previous studies demonstrated that high frequency stimulation of the basolateral amygdaloid nucleus (Bla) elicits LTP in the IC (Escobar et al., 1998a,b; Jones et al., 1999). Moreover, we have shown that induction of LTP in the Bla-IC pathway before CTA training enhances the retention of this task (Escobar and Bermúdez-Rattoni, 2000; Castillo et al., 2006).

Research on the cellular basis of learning and memory has identified some key molecules involved in the processes of acquisition and consolidation of information (Lamprecht and LeDoux, 2004). However, little is known about the processes involved in the permanence of long-term memory (LTM). Recently, the search of molecular mechanisms involved in LTM storage has highlighted a significant participation of calcium/calmodulin dependent protein kinase II (CaMKII). The increase in calcium concentration after LTP induction leads to CaMKII autophosphorylation, an event that makes CaMKII activity persist even after the decay of calcium concentration (Lisman et al., 2002, 2012; Colbran and Brown, 2004). However, it has been demonstrated that this autonomous activity is only transient (Lengyel et al., 2004; Otmakhov et al., 2015; Murakoshi et al., 2017), while Thr286 phosphorylation has been proved as a persistent event, that has been observed up to 8 h after stimulation (Ahmed and Frey, 2005). In this regard, it has been observed that synaptic potentiation in hippocampal CA1 region is reverted when ant-CaMKIINtide, a noncompetitive inhibitor of CaMKII, is applied during the maintenance phase of CA1-LTP *in vitro* (Sanhueza et al., 2007). Similar results have been observed by our research group when CaMKIINtide is infused in CA3 region during the maintenance phase of *in vivo* mossy fiber (MF)-LTP (Juárez-Muñoz et al., 2017). In a recent study it has been proved that expression of a transient dominant-negative form of CaMKII erases a previously stablished hippocampal-dependent memory, pointing to a role of this molecule for stable memory storage (Rossetti et al., 2017). It has also been shown that training in a spatial task elicits increments in hippocampal CaMKII autophosphorylation (Tan and Liang, 1996). Furthermore, intrahipocampal pharmacogenetic inhibition of CaMKII activity impairs retention of spatial memory (Babcock et al., 2005). Importantly, it has been shown that although mice heterozygous for a CaMKII null mutation have normal memory retention for contextual fear and water maze tasks 1–3 days after training, these animals exhibit amnesia when tested 10–50 days post-training (Frankland et al., 2001), revealing a role for CaMKII in the persistence of memory.

Since little is known about the molecular actors implicated in the maintenance of synaptic plasticity and LTM, in the present work we evaluated the role of CaMKII in the maintenance of *in vivo* Bla-IC LTP as well as in the persistence of CTA-LTM.

## Materials and methods

### Animals

Seventy-three male Wistar rats, weighing 360–390 g were prepared for the present study. Rats were individually caged and maintained on a 12:12 light–dark cycle at 22° C with water and food available *ad libitum* except where indicated (Martínez-Moreno et al., 2016). Experiments were performed in accordance with the Norma Oficial Mexicana and with the approval of the Animal care committee of the Faculty of Psychology of the National Autonomous University of Mexico.

### Electrophysiology procedure

Electrophysiological recordings were performed in anesthetized rats as previously described (Escobar et al., 1998a; Rodríguez-Durán et al., 2011; Rivera-Olvera et al., 2016). Briefly, rats were anesthetized with pentobarbital (50 mg/kg i.p.). Responses were recorded by using a monopolar microinfusion electrode placed in the IC. Constant current stimulation (50–70 μA monophasic pulses, 0.25 ms duration) was applied to the Bla unilaterally using a stainless steel bipolar electrode. The microinfusion electrodes were coupled to 10 μl Hamilton syringes (Reno, NV, USA) driven by a microinfusion pump (Cole Parmer Co., Vernon Hills, IL, USA). Evoked responses from IC were measured by recording the EPSP slope. During the 30 min baseline period responses were evoked at 0.05 Hz. LTP was induced by delivering 10 trains of 100 Hz/1 s with an intertrain interval of 20 s. Animals with unclear electrode placement were discarded.

### Western blot

Rats were decapitated and the ipsilateral recorded IC area was microdissected. The tissues were subsequently sonicated into a lysis buffer (50 mM Tris-HCl pH 6.8, 20 mM NaCl, 2 mM EDTA, 10% glycerol, 10% triton) supplemented with 10 mM protease inhibitors (Mini Complete, Roche, Manheim, Germany); as well as with phosphatase inhibitors (50 mM NaF, 4 mM Na3VO4, 10 mM NaPPi). Following sonication, samples were centrifuged at 14,000 rpm for 20 min at 4°C and the supernatant was obtained. Protein concentration was measured using Bradford assay, with bovine serum albumin as standard. An equivalent amount of protein for each sample was resolved in 12% SDS-acrylamide gels; blotted electrophoretically and blocked 90 min in TBST buffer (Tris buffered saline containing 0.01%, Tween-20, pH 7.4) containing 5% non-fat milk (Castillo and Escobar, 2011). Membranes were incubated overnight at 4°C with anti-phospho CaMKII T286/287 antibody (1:1,000, #06-881, Millipore, Darmstadt, Germany) for the detection of phosphorylated form of CaMKII and with anti-CaMKII antibody (1:500, #5306, Santa Cruz, CA, USA) for CaMKII total. The phosphorylated isoforms were normalized to the total isoform as a ratio, which was presented as a percentage value in histograms. We performed densitometry using the software off-line ImageJ (NIH, USA).

### Cannulae implantation

Using a previously described procedure, animals were bilaterally implanted in the IC with stainless steel guide cannulae (Moguel-González et al., 2008; Rodríguez-Serrano et al., 2014). Microinjectors were attached by polyethylene tubing to a 10-μl Hamilton syringe driven by a microinfusion pump (Cole Parmer Co., Vernon Hills, IL, USA). Animals were allowed to recover for 1 week after surgery. Histological analysis was performed on all groups to verify the injector tip location.

### CTA

As previously described (Rivera-Olvera et al., 2016), 7 days after surgery animals were trained to drink water twice a day from a graduated cylinder, during 10 min trials for 3 days. On the acquisition session, water was replaced by a saccharin solution 0.1% (Sigma, St. Louis, MO), and 10 min later, animals were intraperitoneally injected with LiCl (0.15 M; 7.5 ml/kg). During the aversion test 0.1% saccharin solution was presented again after two more days of baseline consumption. The strength of aversion was measured through the reduction of saccharin consumption.

### Experimental design

**Figure 1:**
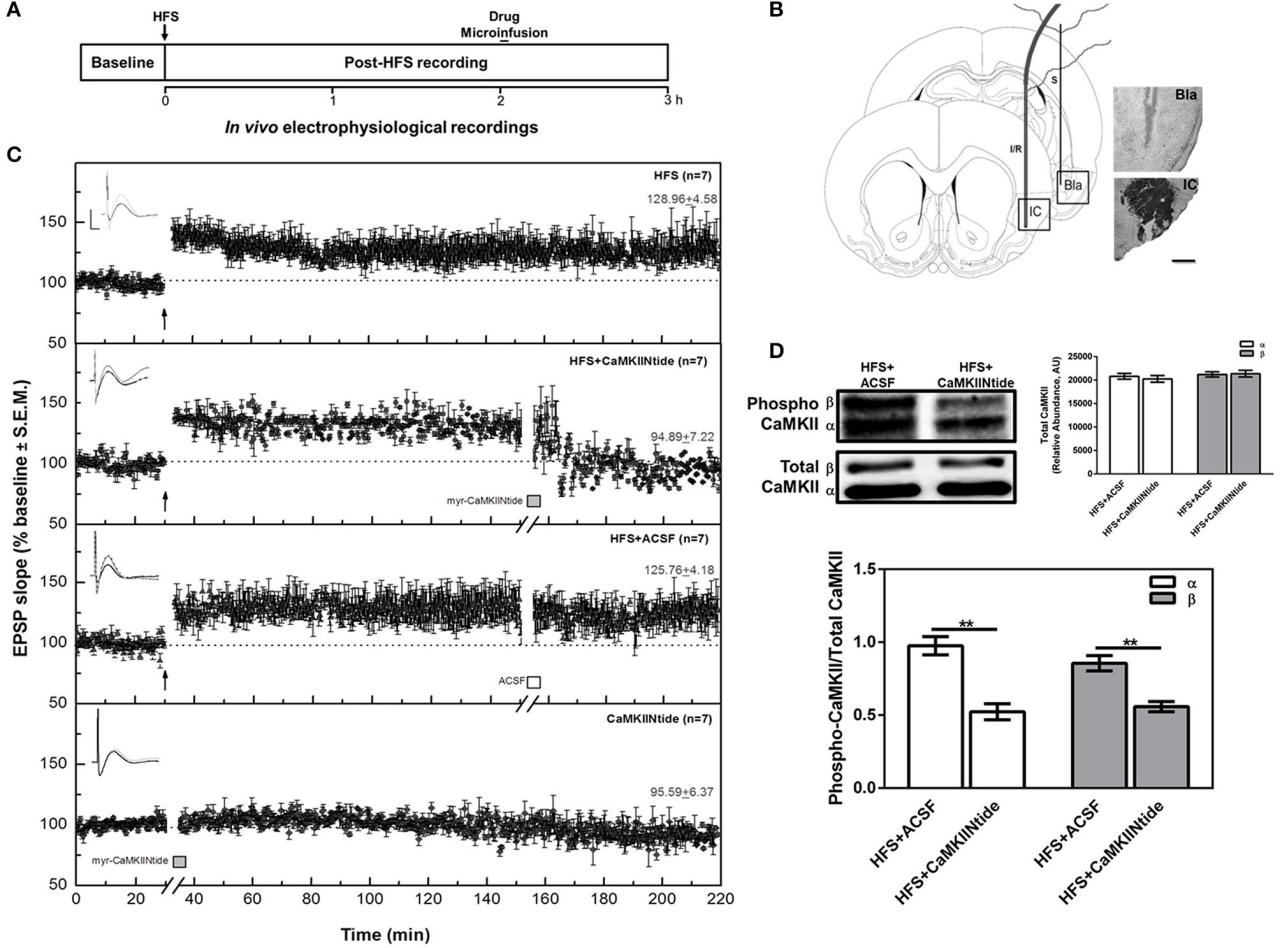
CaMKII inhibition reverts IC-LTP. **(A)** Schematic representation of the experimental procedure. **(B)** Schematic representation and representative micrographs of electrode placement showing the stimulated (S) and infused/recorded (I/R) sites in a coronal plane. The representative microphotography of IC shows the CaMKIINtide diffusion through the combination of the drug with Ponceau red. Bla, basolateral amygdaloid nucleus; IC, insular cortex. Scale bar: 1 mm. **(C)** Plot of IC evoked responses from HFS, HFS+CaMKIINtide, HFS+ACSF and CaMKIINtide groups. Note that the inhibition of CaMKII reverts Bla-IC potentiation. The top of each graph shows representative examples of IC field potentials obtained before (black line), 120 min after HFS (gray line) and 60 min after the infusion (dotted line). Scale bar: 5 ms, 0.5 mV. Bars indicate the infusion period (5 min). Arrows indicate HFS delivery. **(D)** Representative Western blots and densitometric analysis of phosphorylated and total CaMKII from HFS+CaMKIINtide and HFS+ACSF groups. Note that infusion of the inhibitor during IC-LTP maintenance phase decreased phosphorylation of CaMKII. AU, Arbitrary units. ^**^p < 0.01.

To analyze the effect of CaMKII inhibition on the maintenance of Bla-IC-LTP, animals were divided into the following treatment groups: (1) HFS group (*n* = 7), which underwent surgery, had electrodes implanted and received high frequency stimulation (HFS) capable of inducing LTP; (2) HFS+CaMKIINtide group (*n* = 7), which in the same conditions of HFS group received intracortical microinfusion of myr-CaMKIINtide (5 μM/μl ACSF/.02 μl/min; Sanhueza et al., 2007; Juárez-Muñoz et al., 2017) prepared with artificial cerebrospinal fluid (ACSF) as vehicle 2 h after HFS delivery; (3) HFS+ACSF group (*n* = 7) which under the same conditions as the HFS+CaMKIINtide group, received intracortical microinfusion of ACSF (1 μl); (4) CaMKIINtide group (*n* = 7) which had electrodes implanted and without prior manipulation received intracortical microinfusion of myr-CaMKIINtide (5 μM/μl ACSF/0.02 μl/min). In order to analyze the state of phosphorylation of CaMKII in the presence of myrCaMKIINtide, three additional animals from each of HFS+ACSF and HFS+CaMKIINtide groups were decapitated and tissue was obtained 130 min after HFS application (i.e., 10 min after myr-CaMKIINtide administration) (Figure 1A).

**Figure 2:**
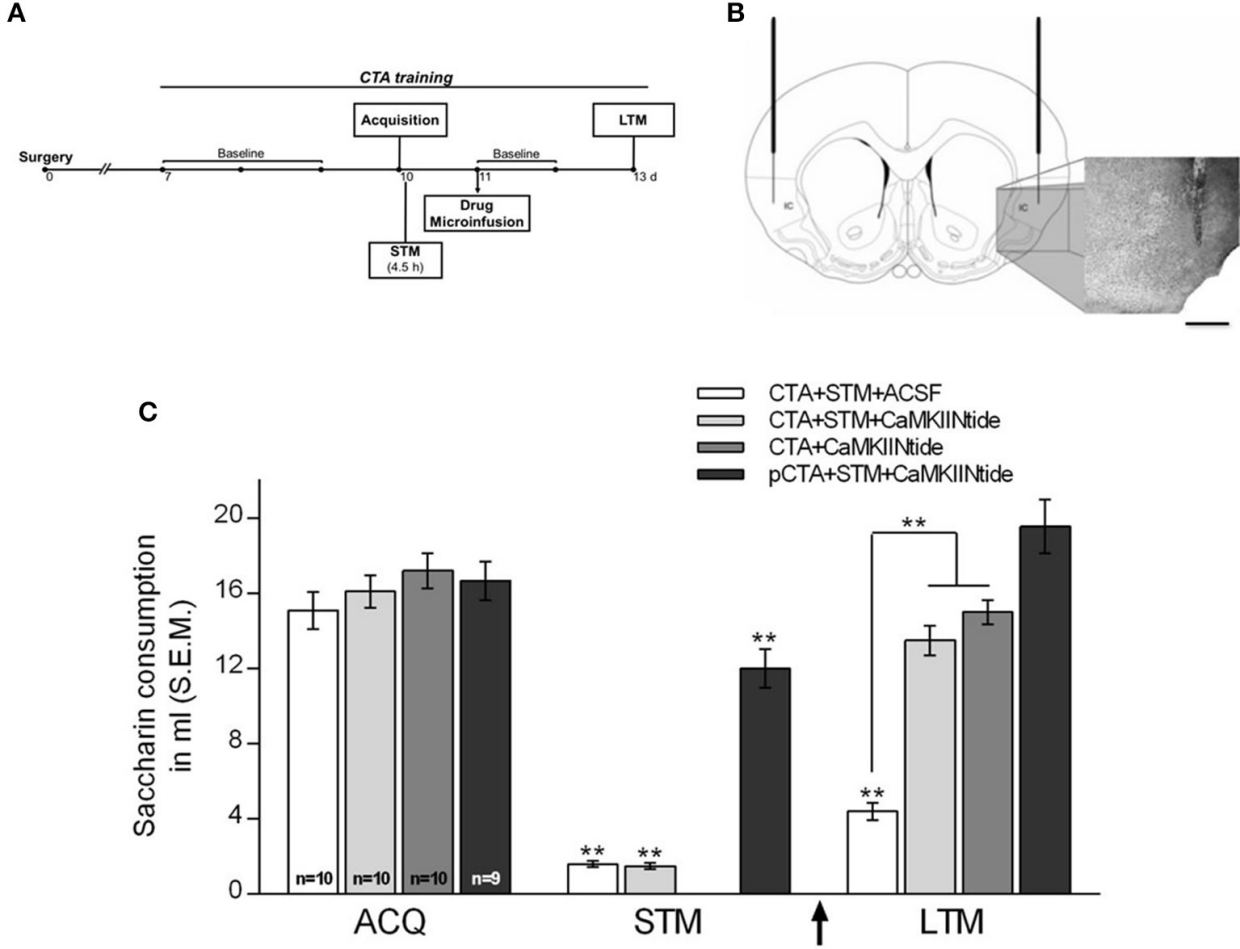
CaMKII inhibition impairs the maintenance of CTA-LTM. **(A)** Schematic representation of the experimental procedure. **(B)** Schematic representation and representative micrographs showing cannulae placement. IC, insular cortex. Scale bar: 1 mm. **(C)** Saccharin consumption during acquisition, short-term memory aversion test (STM) and long-term memory aversion test (LTM) for CTA+STM+ACSF, CTA+STM+CaMKIINtide, CTA+CaMKIINtide and pCTA+STM+CaMKIINtide groups. Note that local infusion of myr-CaMKIINtide in the IC 24 h after acquisition, either in the presence or absence of STM-test produced a severe deficit during the LTM aversion test. ^**^p < 0.001. Arrow indicates microinfusion.

To evaluate the effect of CaMKII inhibition on the maintenance of CTA-LTM, animals were divided into the following treatment groups: (1) CTA+STM+CaMKIINtide group (*n* = 10), which was trained in CTA and was subjected to a short-term memory aversion test (STM) carried out 4.5 h after the acquisition in order to corroborate the association of saccharin with gastric malaise. One day after acquisition rats received bilateral intracortical microinfusion of myr-CaMKIINtide (5 μM/μl ACSF/0.02 μl/min; Sanhueza et al., 2007; Juárez-Muñoz et al., 2017) prepared with artificial cerebrospinal fluid (ACSF). LTM was assessed 48 h after infusion; (2) CTA+STM+ACSF (*n* = 10), which under the same conditions of CTA+STM+CaMKIINtide group received bilateral intracortical microinfusion of ACSF (1 μl); (3) CTA+CaMKIINtide (*n* = 10), which was trained in CTA and received bilateral intracortical microinfusion of myr-CaMKIINtide in absence of STM aversion test in order to prevent the inherent interference to retrieval process (Nader and Einarsson, 2010); (4) pCTA+STM+CaMKIINtide (*n* = 9), which was pseudotrained in CTA (during the conditioning session PBS was delivered instead of LiCl) and received bilateral intracortical microinfusion of myr-CaMKIINtide (Figure 2A).

## Results

### Histology

Histological examinations revealed that the stimulating and recording electrodes were correctly located in the Bla and the IC, respectively, in all animals included in the present analysis (Figure 1B). Similarly, injectors were correctly placed in the IC for all groups (Figure 2B).

### Electrophysiology

The IC EPSP consisted of potentials of 0.47 ± 0.004 mV (mean ± SEM), elicited with 50–70 μA current pulses of 0.1–0.25 ms duration. These responses initiate at 2–3 ms post-stimulation and presented their peak at 7–9 ms with an average slope of 0.019 ± 0.003 (mean ± SEM), in agreement with previous studies (Escobar et al., 1998a, 2002; Jones et al., 1999; Rodríguez-Durán et al., 2011).

### CaMKII is necessary for the maintenance of *in Vivo* HFS induced potentiation of synaptic transmission at Bla-IC pathway of adult rats

As previously described, HFS produced an enhancement in the IC field EPSP slope values, with a duration of at least 3 h (HFS group). The application of myr-CaMKIINtide (5 μg/1 μl) in the IC (HFS+CaMKIINtide group) blocked the maintenance phase of synaptic potentiation when the inhibitor was applied 2 h after HFS (Figure 1C), while infusion of ACSF (1 μl, HFS+ACSF group) had no effect over the maintenance phase of LTP. Remarkably, the application of the inhibitor in the absence of HFS did not have any effect over baseline transmission (CaMKIINtide group). ANOVA analysis for slope increases revealed highly significant group differences [*F*_(3, 24)_ = 71.28; *P* < 0.001]. *Post-hoc* analysis with Fisher's test showed significant differences between the HFS+CaMKIINtide group and all the groups that received HFS (*P* < 0.001). At 1 h post-infusion, the percent changes (±SEM) in the EPSP slope for the HFS, HFS+CaMKIINtide, HFS+ACSF and CAMKIINtide groups were 128.96 ± 4.58, 94.89 ± 7.22, 125.76 ± 4.18, and 95.59 ± 6.37 respectively.

Western blot analysis showed that phosphorylation of the two main cortical isoforms of CaMKII (α and β) were decreased by myr-CaMKIINtide administration. As shown in Figure 1D, the administration of the inhibitor of CaMKII during the maintenance phase of synaptic potentiation decreased CaMKII phosphorylation in the IC region of animals from group HFS+CaMKIINtide, as compared to HFS+ACSF group [Two-Way ANOVA; *F*_(1, 8)_ = 52.00; *p* < 0.05]. No changes were observed in CaMKII total expression.

### CamkII inhibition impairs the maintenance of CTA-LTM

No significant differences were found among groups neither in the baseline water intake nor during the acquisition session. Intracortical microinfusion of myr-CaMKIINtide 24 h after CTA acquisition lead to a memory impairment during LTM aversion test, performed 48 h after the inhibitor microinfusion, i.e., 72 h after the CTA acquisition. Two-way ANOVA revealed significant group differences [*F*_(3, 35)_ = 22.75, *p* < 0.001]. *Post-hoc* analysis with Fisher's test revealed that groups CTA+STM+CaMKIINtide and CTA+CaMKIINtide showed significant differences compared to CTA+STM+ACSF and pCTA+STM+CaMKIINtide groups (*p* < 0.001). During the STM aversion test groups CTA+STM+VEH and CTA+STM+CaMKIINtide showed a significant reduction in the consumption of saccharin solution, confirming that the association with the gastric malaise was well established, as shown in Figure 2C.

## Discussion

Relatively little is known about the LTP maintenance processes that underlie the persistence of synaptic memory. The results of the present study demonstrate that CaMKII is necessary for the maintenance of *in vivo* HFS induced potentiation of synaptic transmission at Bla-IC pathway, considered as an important excitatory circuit implicated in the acquisition and storage of CTA. The intracortical application of the CaMKII inhibitor, myr-CaMKIINtide, 2 h after HFS delivery suppressed the maintenance phase of synaptic potentiation. The suppression can most likely be attributed to a decrease in the CaMKII phosphorylation, as confirmed by the immunoblot analysis. Remarkably, the application of the inhibitor in the absence of HFS did not have any effect over baseline transmission. Previous studies have revealed an important potential role for CaMKII in memory maintenance but most have been performed in the hippocampus. In this regard, Sanhueza and colleagues, have reported that application of an inhibitor of CaMKII lead to the decrement of synaptic potentiation during the maintenance phase of *in vitro* N-methyl-D-aspartate (NMDA) receptors dependent LTP in CA1 region (Sanhueza et al., 2007, 2011), and recently our research group has observed similar results in the hippocampal MF pathway *in vivo* (Juárez-Muñoz et al., 2017).

Regarding the mechanisms, it has been described that autophosphorylation of CaMKII generates autonomous activity, as well as post-synaptic translocation to interact with target proteins, such as α-amino-3-hydroxy-5-methyl-4-isoxazolepropionic acid (AMPA) and NMDA receptors (Strack and Colbran, 1998; Baucum et al., 2015). Indeed, the absence of T286 CaMKII autophosphorylation blocks the hippocampal NMDA dependent LTP (Giese et al., 1998; Yamagata et al., 2009). In addition, it has been well established that autonomous CaMKII phosphorylates AMPA receptors, an event that accounts for LTP induction (Derkach et al., 1999; Ghosh et al., 2015). On the other hand, it has been proposed that the association of CaMKII with GluN2B subunits of NMDA receptors is a central mechanism required for the maintenance phase of LTP in the hippocampus (Sanhueza et al., 2011). CaMKII-GluN2B binding persists even after LTP stimulus decay and this association leaves the kinase in a partially autonomous conformation that mediates its redistribution (Strack et al., 2000; Bayer et al., 2006; O'Leary et al., 2011). Accumulation of CaMKII-GluN2B at the post-synaptic density promotes the capture of multiple proteins including actinin, densin, delta-catenin and N-cadherin, acting as a structural scaffold for a series of binding reactions that together contribute to LTP maintenance (Sanhueza and Lisman, 2013). Moreover, it is known that structural reorganization as a result of activity is required for maintenance of synaptic strength (Lamprecht and LeDoux, 2004; Lynch et al., 2007). In this regard, it has been demonstrated that CaMKII inhibition during the maintenance phase of MF-LTP prevents the activity-dependent morphological reorganization in this pathway (Juárez-Muñoz et al., 2017). In addition, several studies have demonstrated the interaction of CaMKII with actin polymerization (Fink et al., 2003; Kim et al., 2015), as well as with enzymes of the Rho/Rac family controlling spine dynamics, turnover and morphology (Murakoshi et al., 2011).

It is well established that the Bla-IC pathway contributes to the formation and retention of CTA memory (Escobar and Bermúdez-Rattoni, 2000; Rodríguez-Durán et al., 2011, 2017; Rodríguez-Durán and Escobar, 2014). CTA and IC-LTP share similar molecular mechanisms, such as NMDA receptor dependence (Escobar et al., 1998a, 2002; Rodríguez-Durán and Escobar, 2014), activation of extracellular signal-regulated kinase-1/2 (ERK1/2), immediately-early gene expression (Jones et al., 1999), and protein synthesis dependence (Moguel-González et al., 2008; Rodríguez-Durán et al., 2011). However, the identity of the actors involved in CTA-LTM maintenance remains largely unknown. Our present findings reveal that CaMKII is a relevant actor for the maintenance of CTA-LTM, since intracortical microinfusion of myr-CaMKIINtide 24 h after CTA acquisition lead to memory impairment during LTM aversion test, performed 72 h after CTA acquisition. Importantly, the effect of CaMKII inhibition on CTA-LTM was specific to CS-US association, since the memory deficit was not present when rats were pseudotrained in CTA.

In this line of ideas, it has been shown that training rats in the Morris water maze positively correlates with an increase in hippocampal CaMKII autonomous activity (Tan and Liang, 1996). In addition, learning of a step-down inhibitory avoidance task increases hippocampal CaMKII activity (Cammarota et al., 1998). Recently, it has been found that expression of a dominant-negative form of CaMKII lead to a strong reduction in spatial memory of mice that persisted even after this form was no longer expressed (Rossetti et al., 2017). In the case of IC, it has been shown that exposure to a novel taste lead to an increase in CaMKII autophosphorylation for up to 3 h after taste consumption. In addition, application of a selective CaMKII inhibitor in the IC 25 min after saccharine consumption attenuated CTA memory tested 3 or 5 h after the establishment of the association (Adaikkan and Rosenblum, 2015). On the other hand, studies involving genetic disruption of CaMKII activity have shown that homozygous αCaMKII mutant mice present deficits in spatial learning (Silva et al., 1992), however they can be overcome with extended training (Elgersma et al., 2002). This compensation cannot be found in mice with a threonine-to-alanine point mutation of CaMKII, preventing the autophosphorylation of the kinase (Giese et al., 1998; Need and Giese, 2003). Moreover, CaMKII autophosphorylation-deficient mutant mice exhibit impaired *de novo* gene transcription required for contextual fear memory consolidation (von Hertzen and Giese, 2005). In a pioneer study Frankland and colleagues, demonstrated that while mice heterozygous for a CaMKII null mutation have normal memory retention for contextual fear and water maze tasks 1–3 days after training, these animals were amnesic when tested 10–50 days post-training, suggesting a role for CaMKII not only in consolidation but also in the maintenance of memory (Frankland et al., 2001). Since the association between CaMKII and GluN2B has been proposed as a substrate for memory maintenance, mice with a mutation that prevents the formation of the CaMKII/GluN2B complex show memory impairment in the Morris water maze when tested at 1 or 3 days after the last training session (Halt et al., 2012).

In summary, our present results show that CaMKII inhibition in the IC during the late-phase of *in vivo* LTP of the Bla-IC projection, described as a necessary pathway for acquisition and storage of CTA memory, blocks the potentiation maintenance. In the same manner, inhibition of CaMKII in the IC 24 h after CTA acquisition impairs the CTA memory persistence. Together these results indicate that CaMKII is a central key component for the maintenance of neocortical synaptic plasticity as well as for persistence of CTA-LTM.

## Author contributions

YJ-M: Acquisition, analysis, and interpretation of data; drafting the article and revising it critically for important intellectual content. LR-L: Acquisition, analysis, and interpretation of data. ME: Conception and design; analysis and interpretation of data, drafting the article and revising it critically for important intellectual content.

### Conflict of interest statement

The authors declare that the research was conducted in the absence of any commercial or financial relationships that could be construed as a potential conflict of interest.
